# Tumor-suppressor activity of RRIG1 in breast cancer

**DOI:** 10.1186/1471-2407-11-32

**Published:** 2011-01-25

**Authors:** Guihong Zhang, Abenaa Brewster, Baoxiang Guan, Zhen Fan, Powel H Brown, Xiao-Chun Xu

**Affiliations:** 1 Department of Clinical Cancer Prevention, The University of Texas M. D. Anderson Cancer Center, Houston, Texas 77030, USA; 2 Department of Experimental Therapeutics, The University of Texas M. D. Anderson Cancer Center, Houston, Texas 77030, USA; 3 Department of Pathology, Anhui Medical University, Hefei, Anhui, China

## Abstract

**Background:**

Retinoid receptor-induced gene-1 (RRIG1) is a novel gene that has been lost in several types of human cancers. The aim of this study was to determine whether RRIG1 plays a role in breast cancer, such as in the suppression of breast cancer cell growth and invasion.

**Methods:**

Immunohistochemistry was used to detect RRIG1 expression in breast tissue specimens. Gene transfection was used to restore or knock down RRIG1 expression in breast cancer cell lines for analysis of cell viability, colony formation, and migration/invasion potential. Reverse-transcription polymerase chain reaction and western blot assays were used to detect the changes in gene expression. The RhoA activation assay was used to assess RRIG1-induced inhibition of RhoA activity.

**Results:**

The immunohistochemical data showed that *RRIG1 *expression was reduced in breast cancer tissues compared with normal and atypical hyperplastic breast tissues. *RRIG1 *expression was inversely correlated with lymph node metastasis of breast cancer but was not associated with the status of hormone receptors, such as estrogen receptor, progesterone receptor, or HER2. Furthermore, restoration of *RRIG1 *expression inhibited proliferation, colony formation, migration, and invasion of breast cancer cells. Expression of RRIG1 also reduced phosphorylated Erk1/2 and Akt levels; c-Jun, MMP9, and Akt expressions; and RhoA activity. In contrast, knockdown of RRIG1 expression promoted breast cancer cell proliferation, colony formation, migration, and invasion potential.

**Conclusion:**

The data from the current study indicated that *RRIG1 *expression was reduced or lost in breast cancer and that restoration of RRIG1 expression suppressed breast cancer cell growth and invasion capacity. Future studies will determine the underlying molecular mechanisms and define RRIG1 as a tumor-suppressor gene in breast cancer.

## Background

Breast cancer is the leading cause of cancer-related death in women between 35 and 45 years of age and remains the second-leading cause of cancer-related death among all women in the United States [1]. Despite success in screening for early stages of breast cancer and remarkable improvement in treatment outcomes, many women still develop metastatic disease and ultimately die [2-4]. Efforts for ultimate elimination of this disease should include an emphasis on 1) a better understanding of breast cancer biology, including elucidation of the functions of the genes involved in breast cancer development, progression, and metastasis; 2) development of novel biomarkers for early detection, pretreatment staging, prediction of response to treatments, monitoring disease progression, and prognosis of breast cancer; and 3) innovative approaches for treatment and prevention of breast cancer.

Our group recently identified and cloned a novel retinoid receptor-induced gene, RRIG1 [5-8]. We found that the RRIG1 gene covers 4.181 kb of genomic sequences and is localized at chromosome 9q34 with 6 exons, coding a protein with 276 amino acids. RRIG1 mRNA is expressed in a broad range of normal tissues, but its expression is lost in various types of cancers, including breast cancer [5,6]. RRIG1 mediates the effect of RAR-β_2 _on gene expression (e.g., Erk1/2 and COX-2) and cell growth. RRIG1 expression was found to be correlated with tumor differentiation but inversely correlated with lymph node metastasis of esophageal cancer [6]. Furthermore, the transient and stable transfection of a RRIG1 expression vector resulted in growth inhibition of esophageal and prostate cancer cells [5,6,8]. Esophageal cancer cells transfected with RRIG1 also showed reduced tumorigenicity in nude mice [6]. These data strongly indicate that RRIG1 plays an important role in suppressing the development or progression of human cancers [5,6,8]. At the level of signal transduction, expression of RRIG1 inhibited Src phosphorylation and RhoA activation, which is believed to be causally linked to reduced colony formation, invasion, and proliferation in esophageal and prostatic cancer cells. In contrast, transfection of antisense RRIG1 increased RhoA activity and f-actin formation and led to increased colony formation, invasion, and proliferation in these cells [5,8].

In this study, we first determined the correlation of RRIG1 expression in breast tissue specimens with the clinicopathologic characteristics of the patients and then examined the effect of RRIG1 expression on breast cancer growth and invasion. We also explored the changes in expression and phosphorylation or activation of several relevant proteins following experimental elevation or knockdown of RRIG1 expression in breast cancer cells. Our data indicate that RRIG1 may function as a tumor-suppressor gene in breast cancer.

## Methods

### Immunohistochemistry

Paraffin blocks from breast tissue specimens were obtained from the Department of Pathology, Anhui Medical University, Hefei, China, guided by a protocol (#Lab08-015) approved by the institutional review board. Patients gave consent for the use of their tissue specimens in this study. These tissue specimens consisted of samples from 15 normal mammary glands, 9 atypical hyperplasia lesions, 10 cases of ductal carcinoma in situ, and 77 cases of invasive breast cancer. For immunohistochemical analysis, these paraffin sections together with paraffin sections from the organotypic cultures were prepared and stained for RRIG1 as previously reported [6]. Briefly, the tissue sections were deparaffinized twice in xylene for 10 min each and rehydrated in a series of ethanol (100%-50%) and then subjected to antigen retrieval with 0.01 M citric buffer in a pressure cooker for 5 min, followed by blocking of tissue endogenous peroxidase activity with H_2_O_2 _treatment. The processed tissue sections were then incubated with 100 μL of 20% normal goat serum in phosphate-buffered saline (PBS) and anti-RRIG1 antibody (custom-made by Lampire Biological Laboratories, Pipersville, PA, with the CAADGLRKPQVHSARAL peptide as the antigen) at 1:500 dilutions with PBS overnight. The next day, the sections were gently washed three times with PBS and once with PBS containing 0.1% Tween 20. They were then subjected to sequential incubations with a second antibody (goat anti-rabbit IgG; Vector Laboratories, Burlingame, CA) and the ABC solution (in the dark) for 30 min each, with washing between incubations to remove unbound antibodies. The sections were subject to color development with 9-ethylcarbazol-3-amine for 15 min and counterstained with hematoxylin for 1 min. After being covered with a cover slip, the sections were viewed and scored under a microscope. A semiquantitative scoring system was used to score both the staining intensity and the percentage of staining in the tissue sections. The staining intensity was scored as follows: 0, no staining; +, weak staining; ++ positive staining; and +++, very strong staining. The percentage of staining was scored as follows: 0, no staining; +, less than 10% of tumor cells stained; ++, 10-50%; and +++, more than 50% tumor cells stained positive. Both intensity and percentage of staining with 0 or + were considered as negative cases, while both intensity and percentage of staining with ++ and +++ were considered as positive cases.

### Cell lines and culture

The human breast cancer cell lines MCF-7, MDA-MB-231, T47D, SK-Br-3, ZR75-1, and MDA-MB-435 were obtained from ATCC (Manassas, VA) and grown in Dulbecco's modified Eagle's minimal essential medium (DMEM), supplemented with 10% fetal bovine serum, at 37ºC in a humidified atmosphere of 95% air and 5% CO_2_. Esophageal cancer TE-8, the stably RAR-β_2_-transfected TE-8S22, stably RRIG1-transfected TE-8-RRIG1 cells, and prostate cancer PC3 cells were from our previous study [5]. These cell lines were grown in Dulbecco's modified Eagle's minimal essential medium (DMEM), supplemented with 10% fetal bovine serum, at 37ºC in a humidified atmosphere of 95% air and 5% CO_2_. The growth medium for the stable gene transfected cells also contained 200 μg/ml G418 (Invitrogen, Carlsbad, CA).

### RRIG1 expression vector and transient gene transfection

RRIG1 sense and antisense open-reading frames were inserted into pCDNA3.1 plasmid as reported in our previous studies [5,6]. The plasmids were used for transient transfection into MDA-MB-231, MDA-MB-435, and T47D with FuGENE 6 (Roche Diagnostics, Indianapolis, IN) or Lipofectamine™ 2000 (Invitrogen). Thirty-six hours after the transfection, the cells were selected with 1200 μ g/mL G418 (Invitrogen) for 5 days. The total cellular protein was extracted from the cells and subjected to Western blotting analysis. In duplicate experiments, RNA was extracted from the cells and subjected to semiquantitative reverse transcription-polymerase chain reaction (RT-PCR) analysis of gene expression as described previously [6,7]. After preliminary investigation to determine the optimal number of PCR cycles (between 25 and 34), we used 32 cycles of PCR to amplify RRIG1 mRNA. GAPDH was used as a loading control. The primers for RRIG1 mRNA were 5'-CTCCCAGGGTGCCATATTT-3' and 5'-GTCATAGAGCACCCGAGCTT-3', which generated a 211-bp band. The primers for MMP-9 expression were 5'-GCACGACGTCTTCCAGTACC-3' and 5'-GTTTGTATCCGGCAAACTGG-3', which generated a 224-bp band. The primers for SH3GLB2 expression were 5'-GCAGACAGCACCAAGAACTG-3' and 5'-TTTTCAGCTTCTGCCACCTT-3', which generated a 233-bp band. GAPDH primers were 5'-CCCTTCATTGACCTCAACTACATGG-3' and 5'-CATGGTGGTGAAGACGCCAG-3', which generated a 192-bp band.

### MTT assay

The cells were grown and transiently transfected with RRIG1 sense or antisense cDNA and then grown in G418-containing medium for 1 or 5 days. For the methyl thiazolyl tetrazolium (MTT) assay, 20 μL of MTT (5 mg/ml, Sigma, St. Louis, MO) was added to each well of the 96-well plates and incubated for an additional 4 h. After the growth medium was removed, 100 μL of dimethyl sulfoxide was added to the wells to dissolve the MTT crystal, and the optical densities were measured with an automated spectrophotometric plate reader at a single wavelength of 540 nm. The percentage of cell growth was calculated using the formula: % control = ODt/ODc × 100, where ODt and ODc are the optical densities for transfected and vector control cells, respectively.

### Colony formation assay

The ability of cells to form colonies in soft agarose is indicative of anchorage independence and is used as an in vitro criterion of transformation. We examined whether these cells can form colonies in soft agar after gene transfection [9]. Briefly, 2 × 10^3 ^gene-transfected cells were mixed in low-temperature-melting agarose (0.35%) and then placed on top of solidified agarose in 60-mm-diameter dishes. After the top agarose with cells solidified in the cold room for 15 min, the dishes were incubated in a humidified atmosphere of 95% air and 5% CO_2 _at 37°C for 21 days. At the end of the experiments, the colonies were visualized by incubation with MTT at 37°C for 4 h and counted under an inverted microscope at 40× magnification.

### Migration and invasion assay

Boyden chambers coated with and without Matrigel (obtained from BD Biosciences, Bedford, MA) were used for assaying tumor cell invasion and migration ability, respectively [5]. The transfected cells were first starved in medium without fetal calf serum (FCS) overnight, and the cells (5 × 10^4^) were resuspended in the FCS-free medium and put into the top chambers in triplicate. The lower chamber was filled with 10% FCS as the chemoattractant and incubated for approximately 24 h for the migration assay and 48 h for the invasion assay. The surface was then wiped with a cotton swab to remove the cells on the upper surface. The cells that invaded the Matrigel and attached to the lower surface of the filter were fixed and stained with 1% crystal violet solution. The membranes with or without Matrigel were then gently removed from the chamber and mounted on glass slides. Six microscopic fields (at 100× magnification) per chamber were photographed. The cells in the photographs were then counted, and the data were summarized as means ± standard deviation and presented as the percentage of control. Student *t*-test was used to determine statistical differences between the control and RRIG1 sense- or antisense-transfected breast cancer cells.

### Protein extraction and western blotting

Total cellular protein was isolated from the gene-transfected breast cancer cells as described previously [5-7]. The protein concentration was then measured with a Bio-Rad Protein Assay Kit II (BioRad Laboratories, Hercules, CA) according to the manufacturer's protocol. Samples containing 50 μg of protein from control or treated cells were separated by 10-14% polyacylamide sodium dodecyl sulfate-polyacrylamide gel electrophoresis gels and then transferred electrophoretically to a Hybond-C nitrocellulose membrane (GE-Healthcare, Arlington Heights, IL) at 500 mA for 2 h at 4°C. The membrane was subsequently stained with 0.5% Ponceau S (Sigma) containing 1% acetic acid to confirm that proteins were loaded equally and to verify transfer efficiency. The membranes were next incubated overnight in a blocking solution containing 5% bovine skim milk and 0.1% Tween 20 in PBS at 4°C. The next day, the membranes were incubated with primary antibodies for 2 h at room temperature. The antibodies used were anti-p-Erk1/2, t-Erk1/2, p-Akt, t-Akt, p-Stat3, t-Stat3, p-Rb, and Rb (Cell Signaling Technology, Beverly, MA); anti-c-Jun and E2F-1 (Santa Cruz Biotechnology, Santa Cruz, CA); and anti-β-actin (Sigma). The membranes were washed in PBS and incubated for 1.5 h with horse-anti-mouse or goat-anti-rabbit secondary antibody (GE-Healthcare) diluted 1:5000. The membranes were then incubated with enhanced chemiluminescence solution (GE-Healthcare) for 1-2 min and exposed to X-ray film. The target band of proteins from western blots was quantified by using NIH ImageJ 1.34s software (NIH, Bethesda, MD) and normalized to β-actin. After that, the percentage of control was calculated using the formula: value of the RRIG1 sense or antisense transfected cells/value of the vector-only transfected cells.

### RhoA activation assay

The RRIG1 sense and antisense cDNA-transfected breast cancer cells were detached with 0.05% trypsin, counted, reseeded, and cultured in new dishes in DMEM with 10% FCS for 16 hours and then in DMEM without FCS for an additional 12 hours. To activate RhoA, the cells were cultured in DMEM with 10% FCS for 6 h, and the total cellular protein was extracted in an ice-cold lysis buffer containing 20 mM Tris-HCl (pH 7.5), 10 mM MgCl_2_, 150 mM NaCl, 1 mM Na_2_EDTA, 1 mM EGTA, 1% Triton X-100, 2.5 mM sodium pyrophosphate, 1 mM β-glycerophosphate, 1 mM Na_3_VO_4_, and 1 μg/ml leupeptin. Activated GTP-bound Rho protein in the cell lysates was pulled down using a recombinant GST-tagged Rhotekin Rho-binding domain (Millipore, Billerica, MA) and analyzed in western blots using anti-RhoA antibody (Santa Cruz Biotechnology). Levels of the activated RhoA protein were normalized with the total cell lysates that were not subjected to the pulldown assay.

## Results

### Immunohistochemical analysis of *RRIG1 *expression in breast tissue specimens

In this study, we first verified the specificity of our rabbit polyclonal anti-RRIG1 antibody using three different experiments. Briefly, we first performed western blot with this antibody to detect expression of RRIG1 protein in vitro translated with an in vitro translation kit from Promega (Madison, WI). The data showed that this antibody specifically recognized RRIG1 protein (Additional Figure S1a). Meanwhile, in two different cancer cell lines we detected RRIG1 expression, which had been established by Northern blot in our previous study. We found that RRIG1-positive esophageal cancer cell line TE-8S22 expressed RRIG1 protein, whereas the PC3 cells did not show the target band, although the western blot showed a number of nonspecific bands (Additional Figure S1b), indicating that this antibody is not suitable for western blot assay.

**Figure 1:**
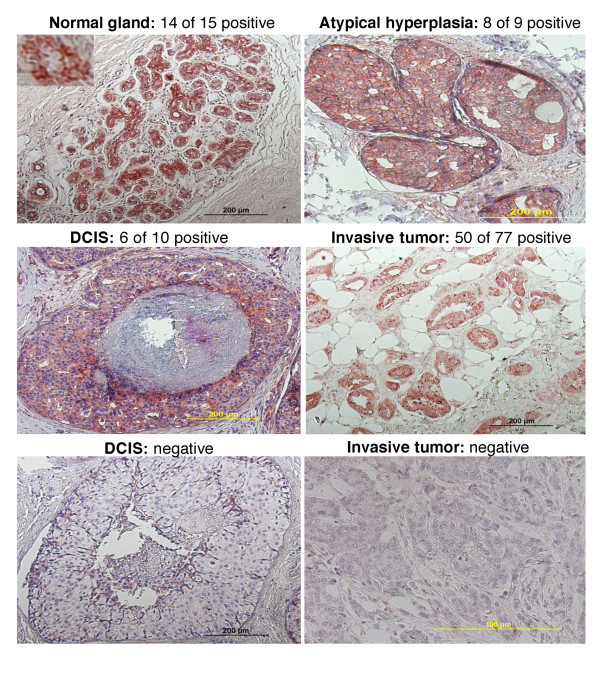
**Immunohistochemical analysis of RRIG1 expression**. Breast tissue sections were immunostained with the rabbit polyclonal anti-RRIG1 antibody. DCIS, ductal carcinoma in situ.

To determine whether this antibody could be used for immunohistochemistry, we compared the data between immunohistochemistry and in situ hybridization for expression of RRIG1 mRNA and protein in the matched cells and tissues (Additional Figure S1cd). These data suggest that this antibody is suitable for immunohistochemistry. Next, we used immunohistochemical analysis to determine the *RRIG1 *expression in breast tissue specimens and found that *RRIG1 *was expressed in 14 of 15 normal mammary glands, 8 of 9 cases of atypical hyperplasia of the mammary gland, 6 of 10 ductal carcinoma *in situ *tissues, and 50 of 77 invasive breast cancer tissues (*P *= 0.023 between normal and invasive cancer by Fisher exact test) (Figure 1).

Finally, we associated RRIG1 expression with the clinicopathologic features of the breast cancer patients and found a statistically significant reverse correlation between *RRIG1 *expression and lymph node metastasis in breast cancer (Table 1).

**Table 1:** Association of RRIG1 expression with clinicopathologic features of breast cancer patients

Variable	RRIG1 expression		
	
	Positive	Negative	p Value
Patient age (years)			0.30
≤ 35	3	0	
35-55	34	17	
> 55	13	10	
Tumor size (cm)			0.66
≤ 2	8	3	
2-5	32	20	
> 5	10	4	
Lymph node metastasis			0.01
+	22	20	
_	28	7	
Tumor differentiation			0.18
I	16	5	
II	27	14	
III	7	8	
Estrogen receptor			0.64
+	25	15	
-	25	12	
Progesterone receptor			
+	27	15	0.89
-	23	12	
c-erbB-2			0.62
+	14	9	
-	36	18	

### RRIG1 regulation of breast cancer cell growth, migration, and invasion

**Figure 2:**
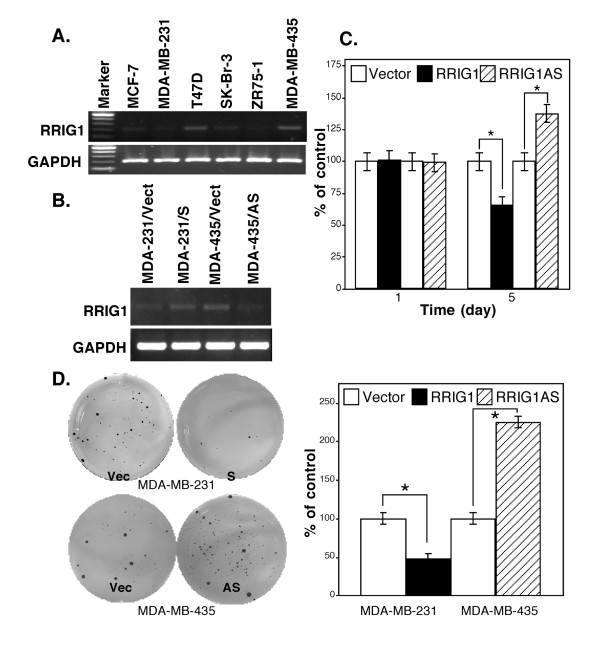
**RRIG1 expression and modulation of breast cancer cell growth and colony formation**. All experiments were repeated once with similar results. **A**, Semiquantitative RT-PCR. Breast cancer cell lines were grown in monolayer, and RNA was then isolated from the cells and subjected to semiquantitative RT-PCR analysis of RRIG1 expression. **B**, Semiquantitative RT-PCR.pCDNA3.1 carrying RRIG1 sense and antisense cDNA was transiently transfected into MDA-MB-231 and MDA-MD-435 cells, respectively. The vector-only plasmid was used as a control. The cells were grown in G418-containing medium, and RNA from the cells was isolated and subjected to semiquantitative RT-PCR analysis. **C**, Cell viability assay. The gene-transfected cells were grown in G418-containing medium for 1 or 5 days, and cell viability was measured using the MTT assay. **D**, Colony formation assay. The gene-transfected cells were grown in soft agar with G418-containing medium for 21 days, and cell colonies were then visualized by incubation with MTT, counted, and summarized. *p < 0.05 vs. the control.

**Figure 3:**
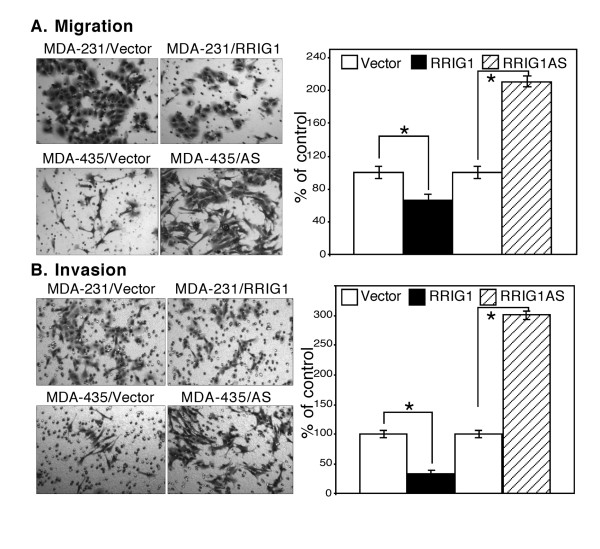
**Tumor cell migration and invasion assays**. All experiments were performed in triplicate and repeated twice. **A**, Migration assay. The RRIG1 sense and antisense-transfected MDA-MB-231 and MDA-MB-435 cells, respectively, were grown in G418-containing medium and then subjected to cell migration assay in Boyden chambers without Matrigel for 24 h. The cells that migrated were stained with 1% crystal violet solution and then counted and summarized. **B**, Invasion assay. These gene-transfected breast cancer cells were grown in G418-containing medium and then subjected to the cell invasion assay in Boyden chambers with Matrigel for 48 h. The invaded cells were stained with 1% crystal violet solution and then counted and summarized. *p < 0.05 vs. the control.

We then detected expression of RRIG1 mRNA in 6 different breast cancer cell lines using semiquantitative RT-PCR and found that MDA-MB-435 and T47D expressed high levels of RRIG1 mRNA and that MCF-7 and SK-Br3 expressed low levels of RRIG1 mRNA. In contrast, MDA-MB-231 and ZR75-1 did not express RRIG1 mRNA (Figure 2A). Given these findings, we chose MDA-MB-231 and MDA-MB-435 for modulation of RRIG1 expression to assess the changed cell behaviors and gene expression. We transiently transfected RRIG1 sense and antisense cDNAs into the MDA-MB-231 and MDA-MB-435 cell lines, respectively. After gene transfection, RRIG1 expression was knocked down by RRIG1 antisense vector in MDA-MB-435 cells, while RRIG1 cDNA transfection restored RRIG1 expression in MDA-MB-231 cells (Figure 2B). Next, we used the MTT assay to detect the changed cell viability by RRIG1 and found that restoration of RRIG1 expression reduced cell viability in MDA-MB-231 cells, but knockdown of RRIG1 expression induced cell viability in MDA-MB-435 cells after 5 d of cultures (Figure 2C). Furthermore, RRIG1 expression decreased the number of colonies in MDA-MB-231 cells, and knockdown of RRIG1 expression in MDA-MB-435 cells increased the numbers of colonies in soft agar (Figure 2D). In addition, our data also showed that RRIG1 decreased migration and invasion capacity of MDA-MB-231 cells but increased the migratory and invasion capacity of MDA-MB-435 cells (Figure 3).

### *RRIG1 *regulation of gene expression in breast cancer cells

**Figure 4:**
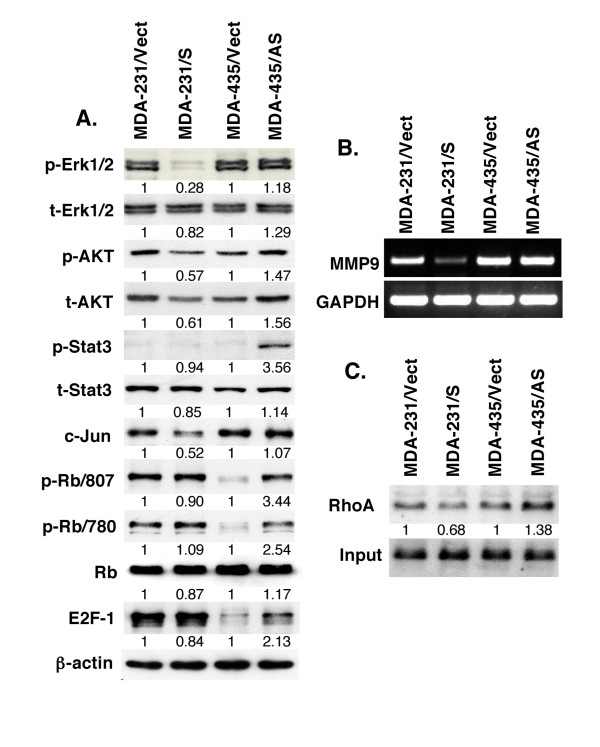
**RRIG1 regulation of gene expression and activities**. All the experiments were repeated at least once with similar results. **A**, Western blotting. The RRIG1 sense and antisense-transfected MDA-MB-231 and MDA-MB-435 cells, respectively, were grown in G418-containing medium for 5 days and then subjected to protein extraction and western blotting analysis of gene expression. The value shown is the percentage of control, which was calculated using the formula: value of the RRIG1 sense or antisense transfected cells/value of the vector-only transfected cells after intensity of the target band of proteins from western blots was quantified by using NIH ImageJ 1.34s software and normalized to β-actin. **B**, Semiquantitative RT-PCR. The gene-transfected cells were grown in G418-containing medium for 5 days, and RNA was then isolated from the cells and subjected to semiquantitative RT-PCR analysis of MMP9 expression. **C**, RhoA activation assay. The gene-transfected cells were grown in G418-containing medium for 5 days and then subjected to the RhoA activation and western blot assays. The data were quantified using the NIH ImageJ software.

**Figure 5:**
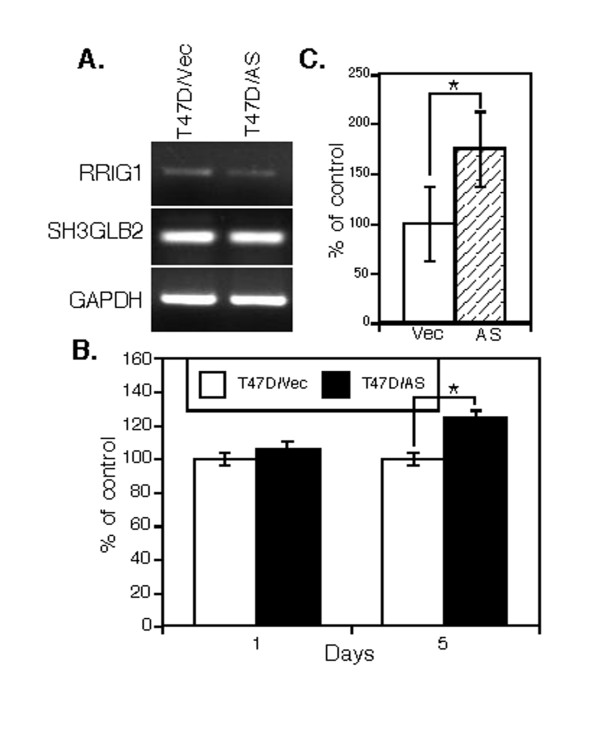
**The effects of antisense RRIG1 in breast cancer T47D cells**. All experiments were repeated at least once with similar results. **A**, Semiquantitative RT-PCR. The gene-transfected cells were grown in G418-containing medium for 5 days, and RNA was then isolated from the cells and subjected to semiquantitative RT-PCR analysis of RRIG1, SH3GLB2, and GAPDH expression. **B**, MTT assay. The gene-transfected cells were grown in G418-containing medium for 1 or 5 days, and cell viability was measured using the MTT assay. **C**, Invasion assay. These gene-transfected breast cancer cells were grown in G418-containing medium and then subjected to the cell invasion assay in Boyden chambers with Matrigel for 48 h. The invaded cells were stained with 1% crystal violet solution and then counted and summarized. Vec, vector-only; AS, antisense RRIG1 cDNA; *p < 0.05 vs. the control.

To determine the underlying molecular events of *RRIG1 *in the inhibition of breast cancer cell proliferation, migration and invasion, we performed semiquantitative RT-PCR and western blotting analysis to assess RRIG1 regulation of gene expression and the RhoA activation assay to detect the changed RhoA activity. We found that RRIG1 expression downregulated p-Erk1/2, p-AKT, total AKT, c-Jun, and MMP9 expression in MDA-MB-231 cells, whereas knockdown of RRIG1 expression upregulated p-AKT, total AKT, p-Stat3, p-RB, and E2F-1 expression in MDA-MBN-435 cells (Figure 4). However, these data demonstrated that RRIG1 sense and antisense cDNAs regulated expression of different genes, indicating that different breast cancer cell lines may have different gene alterations and that RRIG1 can regulate expression of some of them for control of breast cancer cell proliferation, migration, and invasion. Furthermore, we confirmed that RRIG1 was able to suppress RhoA activity, whereas antisense RRIG1 promoted RhoA activity in these breast cancer cells (Figure 4C). Because a recent study reported that the MDA-MB-435 cell line may have originated from melanoma cells [10,11], we added another RRIG1-positive cell line for antisense RRIG1 transfection. Antisense RRIG1 cDNA reduced RRIG1 mRNA levels but did not affect expression of SH3 domain GRB2-like endophilin B2 (SH3GLB2) (Figure 5). Knockdown of RRIG1 expression enhanced cell viability and invasion capacity of T47D cells (Figure 5).

## Discussion

In the current study, we analyzed RRIG1 expression in breast tissue specimens and then determined the role of RRIG1 in breast cancer cells. We found that *RRIG1 *expression is reduced in breast cancer tissues, as reported by us previously [7]. *RRIG1 *expression was inversely correlated with lymph node metastasis of breast cancer. Furthermore, the restoration of *RRIG1 *expression in a breast cancer cell line resulted in tumor-suppressor activities, such as inhibition of tumor cell proliferation, colony formation, migration, and invasion. At molecular levels, RRIG1 suppressed expression of p-Erk1/2, p-Akt, total Akt, c-Jun, and MMP9 and RhoA activity. These genes are related to cell growth, adhesion, and mobility. However, knockdown of RRIG1 expression in another breast cancer cell line promoted tumor cell proliferation, colony formation, migration, and invasion, but these effects were associated with regulation of p-Stat3, p-Rb, and E2F-1.This study suggests that *RRIG1 *plays a role in suppressing breast cancer progression or even development of breast cancer.

Our previous studies [5-7] demonstrated that RRIG1 is a novel gene and may be a putative tumor suppressor. *RRIG1 *gene contains 6 exons, with exons 2, 3, and 5 shared with exons 8, 9, and 10 of SH3GLB2 cDNA [7]. However, the open reading frames of these two genes are different, and therefore they are different genes. Moreover, our study also revealed that these two genes were differentially expressed in esophageal and prostate cancer cell lines [5,8] and that benzo[a]pyrene diol epoxide (BPDE) reduced RRIG1 expression but did not alter SH3GLB2 mRNA levels in esophageal cancer cell lines (our unpublished data). These findings indicate that RRIG1 is a novel protein with no similarities to other proteins and that RRIG1 protein contains several putative functional motifs, such as a cadherin signature-like motif, a glycoprotein GG/GX motif, and proline-rich regions that contain SH3 domain-binding motifs (PxxP). The latter motif was able to functionally mediate RRIG1 antitumor activity by suppressing tumor cell viability and cyclin D1 expression [7]. Nevertheless, it is unclear whether knockdown of RRIG1 expression using a vector carrying antisense open reading frame of RRIG1 can block expression of SH3GLB2 protein (Because no anti-SH3GLB2 antibody is available, we could not perform such an experiment). However, our current study showed that the vector carrying antisense open reading frame of RRIG1 did not affect expression of SH3GLB2 mRNA (Figure 5).

Our current and previous studies clearly demonstrated that reduced or lost expression of *RRIG1 *is an important event in the development or progression of human cancers, although the mechanism underlying the loss of expression of the RRIG1 gene remains unknown [5-8]. Moreover, our previous studies [12-14] reported that benzo[*a*]pyrene diol epoxide (BPDE), a carcinogen present in tobacco smoke and environmental pollution, as well as bile acid, a tumor promoter in the gastrointestinal tract, inhibited RRIG1 expression, whereas retinoic acid induced RRIG1 expression, which was associated with downregulation of RAR-β_2 _expression by these carcinogens. In addition, previous studies demonstrated a loss of RAR-β_2 _expression in breast cancer tissues and cells [15-17]. Further study will be needed to determine the molecular mechanism that contributes to the loss of RRIG1 expression and, most importantly, to define RRIG1 as a tumor-suppressor gene in the inhibition of tumor cell growth, invasion, and gene expression.

Our current study demonstrated that the restoration of *RRIG1 *expression inhibited breast cancer cell proliferation, colony formation, migration, and invasion and regulated gene expression. Inhibition of phosphorylated Erk1/2 and Akt and c-Jun expression by RRIG1 may mediate RRIG1 suppression of cell growth, while reduced MMP9 expression by RRIG1 mediates the reduced tumor cell mobility and invasion capacity. Our previous study showed that MMP9 was upregulated in breast cancer [18]. Erk1/2 and Akt proteins play an important role in cell survival, and activation of Akt has been shown to overcome cell cycle arrest in G1 and G2 phases of the cell cycle to enable cell proliferation [19-22]. Nevertheless, knockdown of RRIG1 expression has had opposite effects in the regulation of breast cancer cell growth, colony formation, migration, and invasion. Interestingly, these effects were through RRIG1 regulation of some other gene expressions, such as phosphorylated Stat3 and Rb. A previous study has shown the retinoic acid suppressed Stat3 phosphorylation in skin cancers [23]. Stat3 is a transcriptional factor and mediates expression of a variety of genes in response to cell stimuli and thus plays a key role in many cellular processes such as cell growth and apoptosis [24,25]. RRIG1 protein contains two putative Src homology 3 (SH3) domain-binding motifs that were characterized in a previous study [26], and our preliminary data showed that RRIG1 was able to bind to Src protein [8]. Previous studies [see reviews in ref. [25]] demonstrated that the role of STAT3 as a downstream signal transducer in Src family kinase-mediated tumorigenesis suggests that RRIG1 may suppress Stat3 activity through binding to Src protein. However, this needs further investigation.

Furthermore, our current study also confirmed our previous finding that RRIG1 inhibits RhoA activation to execute some of its biological functions [5]. RhoA is a small GTPase protein known to regulate the actin cytoskeleton in controlling cell mobility and growth and modulating gene expression [27-31]. The aberrant activation of RhoA proteins was found to cause cell growth, transformation, invasion, and metastasis in experimental models of carcinogenesis, and inhibition of RhoA suppressed cell proliferation, invasion, and angiogenesis in vitro and in vivo [27-31]. The RRIG1 protein can bind to the RhoA protein, although it is unknown whether this binding is direct or indirect [5]. We studied the RRIG1 and RhoA association because, in our unpublished findings of an in vitro pull-down assay, RRIG1 protein was able to pull down RhoA protein.

## Conclusion

Our current study clearly showed the importance of lower *RRIG1 *expression in breast cancer cell growth, colony formation, invasion, and altered gene expression. Future study will attempt to determine the underlying molecular mechanisms and define RRIG1 as a tumor-suppressor gene in breast cancer.

## Competing interests

The authors declare that they have no competing interests.

## Authors' contributions

GZ performed most of the experiments. AB helped in research design and immunohistochemistry. BG performed experiments for T47D cells. ZF provided breast cancer cell lines and contributed to the research design, critical comments, and writing. PHB provided financial support and contributed to the research design. XCX participated in the overall research design, data collection, and writing. All authors read and approved the final Manuscript.

## Pre-publication history

The pre-publication history for this paper can be accessed here:

http://www.biomedcentral.com/1471-2407/11/32/prepub

## Supplementary Material

Additional File 1 **Figure S1: Specificity of rabbit polyclonal anti-RRIG1 antibody for immunohistochemistry**. **A**, Western blot. RRIG1 protein was first in vitro translated with pGEM and pcDNA3.1 vectors-carrying RRIG1 open-reading frame cDNA and an in vitro protein translation kit. The samples from the in vitro translation were subjected to western blot analysis of RRIG1 expression using our polyclonal anti-RRIG1 antibody. NC, negative control. **B**, Western blot. Esophageal cancer TE-8S22 and prostate cancer PC3 cells were grown on monolayer for 3 days and total cellular protein was extracted and subjected to western blot with the polyclonal anti-RRIG1 antibody. The vector controlled in vitro translational sample was used for negative control. NC, negative control. NS, non-specific. **C**, Immunohistochemistry. RRIG-1-negative esophageal cancer cells TE-8 and RRIG-1 stably transfected TE-8-RRIG1 cells were grown in organotypic cultures for 14 days and the 3-D cell layers were subjected to tissue processing and immunohistochemistry with the polyclonal anti-RRIG1 antibody. **D**, Expression of RRIG1 mRNA and protein in breast tissues using in situ hybridization (ISH, see ref. 5 for the detailed methodology) and immunohistochemistry (IHC). **Additional Method Organotypic culture**. Esophageal cancer cell lines TE-8 and TE-8-RRIG1 were seeded on to collagen gels prepared by using type I rat-tail collagen (Collaborative Biomedical Products, Bedford, MA), and incubated overnight until the cells will be confluent. The gels were then elevated to the air-liquid interface by placing them onto a surgical stainless steel mesh platform. DMEM was added until it reached the undersurface of the collagen gel but not cover the cell layer. The cultures were incubated at 37°C for 2 weeks after elevation to the air-medium interface. In the end of the experiments, the cell discs were fixed in 4% paraformaldehyde and embedded in paraffin following by sectioning and staining. Click here for file
