# Supplementary figures and images for: Tumor-suppressor activity of RRIG1 in breast cancer
Source: BMC Cancer. 2011 Jan 25;11:32. doi: 10.1186/1471-2407-11-32 (PMC3037341; doi:10.1186/1471-2407-11-32)

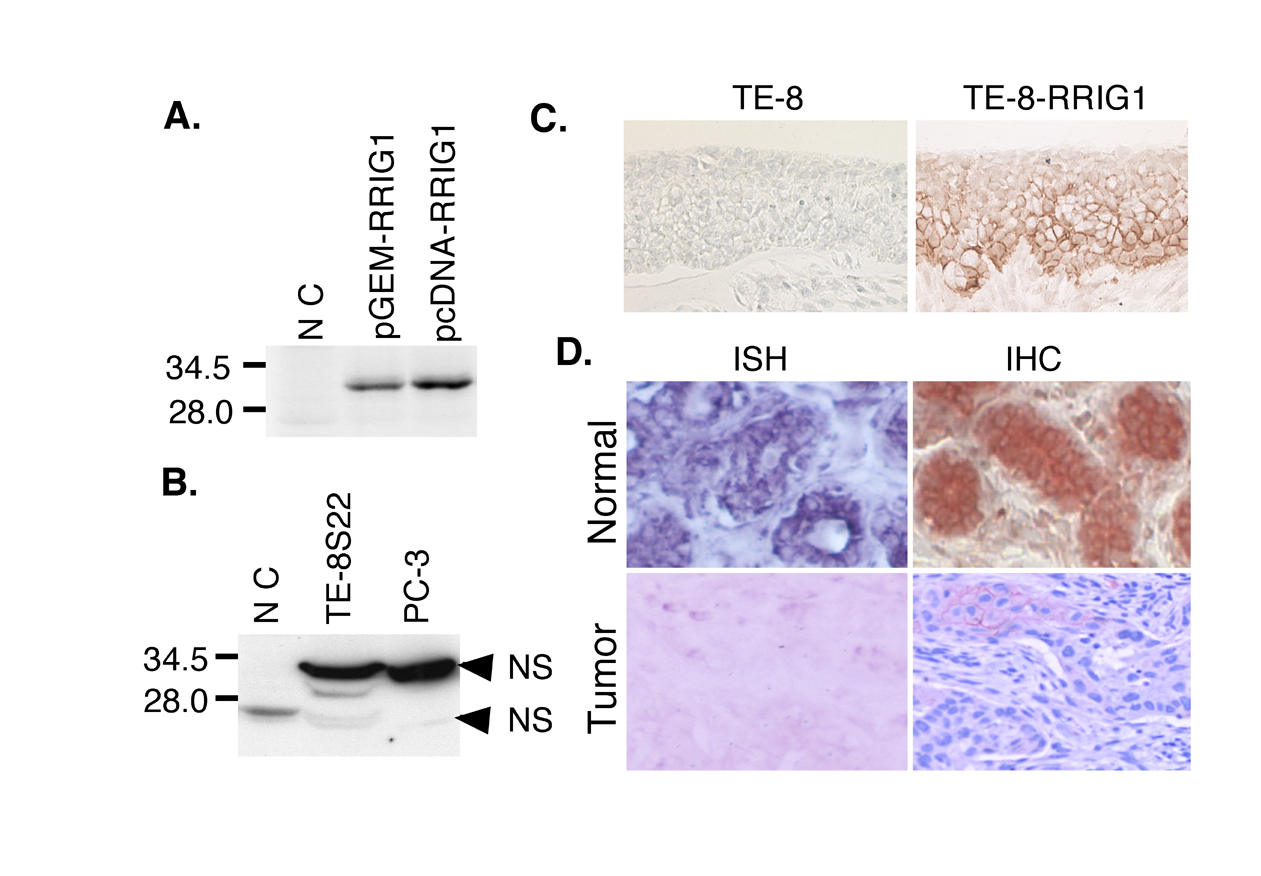

Supplement: Additional File 1 — Figure S1: Specificity of rabbit polyclonal anti-RRIG1 antibody for immunohistochemistry. A, Western blot. RRIG1 protein was first in vitro translated with pGEM and pcDNA3.1 vectors-carrying RRIG1 open-reading frame cDNA and an in vitro protein translation kit. The samples from the in vitro translation were subjected to western blot analysis of RRIG1 expression using our polyclonal anti-RRIG1 antibody. NC, negative control. B, Western blot. Esophageal cancer TE-8S22 and prostate cancer PC3 cells were grown on monolayer for 3 days and total cellular protein was extracted and subjected to western blot with the polyclonal anti-RRIG1 antibody. The vector controlled in vitro translational sample was used for negative control. NC, negative control. NS, non-specific. C, Immunohistochemistry. RRIG-1-negative esophageal cancer cells TE-8 and RRIG-1 stably transfected TE-8-RRIG1 cells were grown in organotypic cultures for 14 days and the 3-D cell layers were subjected to tissue processing and immunohistochemistry with the polyclonal anti-RRIG1 antibody. D, Expression of RRIG1 mRNA and protein in breast tissues using in situ hybridization (ISH, see ref. 5 for the detailed methodology) and immunohistochemistry (IHC). Additional Method Organotypic culture. Esophageal cancer cell lines TE-8 and TE-8-RRIG1 were seeded on to collagen gels prepared by using type I rat-tail collagen (Collaborative Biomedical Products, Bedford, MA), and incubated overnight until the cells will be confluent. The gels were then elevated to the air-liquid interface by placing them onto a surgical stainless steel mesh platform. DMEM was added until it reached the undersurface of the collagen gel but not cover the cell layer. The cultures were incubated at 37°C for 2 weeks after elevation to the air-medium interface. In the end of the experiments, the cell discs were fixed in 4% paraformaldehyde and embedded in paraffin following by sectioning and staining. [file 1471-2407-11-32-S1.TIFF]
